# Supplementary material for: Carbonate complexation enhances hydrothermal transport of rare earth elements in alkaline fluids
Source: Nat Commun. 2022 Mar 18;13:1456. doi: 10.1038/s41467-022-28943-z (PMC8933457; doi:10.1038/s41467-022-28943-z)
Supplement: Supplementary file 1 — Supplementary Materials [file 41467_2022_28943_MOESM1_ESM.pdf]

Supplementary Materials

Figure S1: (A) XANES for Gd in 0.7m Na_2CO_3 and 0.7m Na_2CO_3-0.3m NaF solutions at 200°C and 80 MPa. (B) XANES for Yb in 0.7m Na_2CO_3 and 0.7m Na_2CO_3-0.3m NaF solutions at 200-300°C and 80 MPa, compared to that of Yb in 0.1mCl at 200°C and 80 MPa. The dashed lines underline differences between alkaline and HCl solutions.

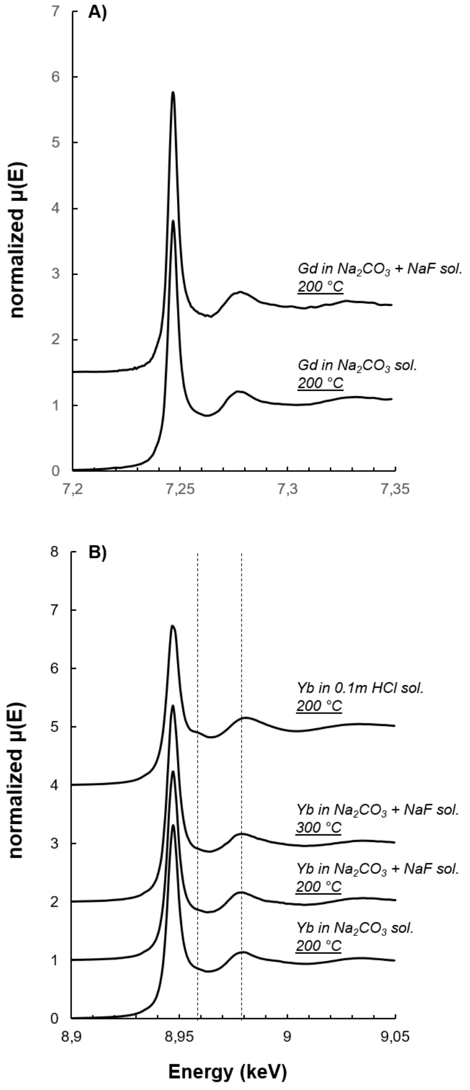

Figure S2: Evolution of the fluorescence eH value as a function of decreasing density (ie., increasing temperature at constant pressure) for three different reference solutions containing respectively 1wt% La in 5m Cl_{tot}, 200ppm Gd + 200ppm Yb in 0.1m HCl and 8500ppm Yb in 0.1m HCl. The stability of eH values from >1 to ~ 0.7g.cm^{-3} suggest that eH values is directly proportional to the REE concentration in hydrothermal fluids. The strong decrease of eH values for $\rho < 0.7$g.cm^{-3} is accompanied by precipitation of REE solids and thus cannot be attributed to a sole effect of density on the fluorescence signal, but rather to the decrease of REE solubility as supercritical conditions are approached.

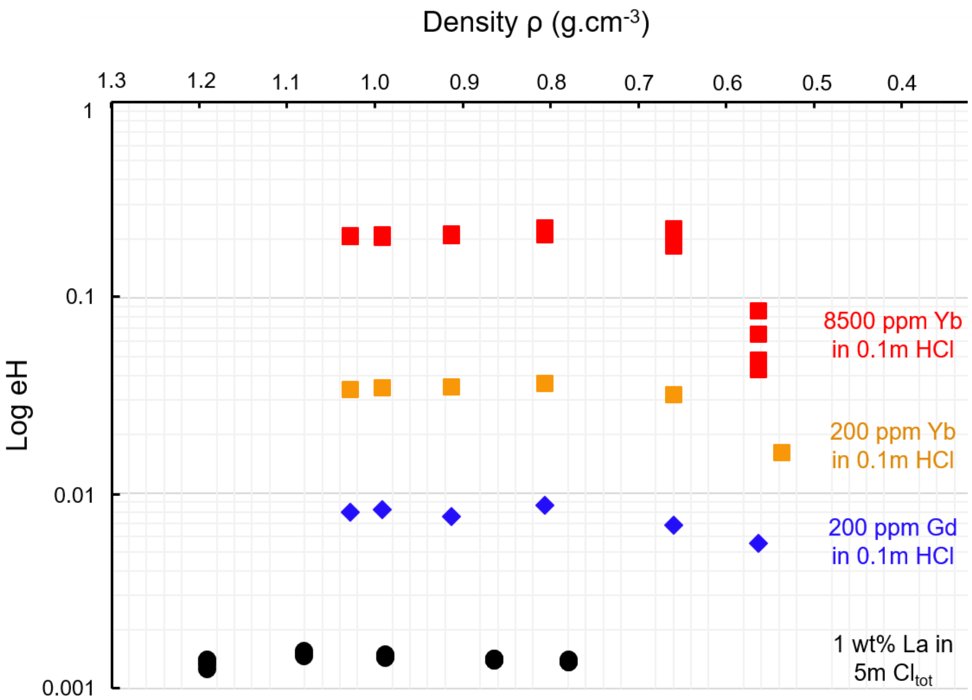

Table S1: Summary of investigated compositions under near neutral to basic conditions and available solubility/speciation results.

Fluid composition and available ligands	Investigated REE	$pH_{25°C}$	Solubility /EXAFS
0.75m LiCl	Er	7	bdl/na
0.5m NaF	Yb	7	bdl/na
0.1-4m NaOH	Sm, Yb, Y	>12	bdl/na
0.5m NaOH – 0.5mNaF	Yb	>12	bdl/na
1mNaOH –3.8m NaF	Sm	>12	>bdl/na
0.5m NaOH – 2m NaCl	Y	>12	bdl/na
0.05m Na_2CO_3	Yb	11.6	bdl/na
0.7m Na_2CO_3	La, Gd, Yb	12	C_i/EXAFS
0.7m Na_2CO_3 + 0.3-0.35m NaF	La, Gd, Yb	12	C_i/EXAFS
0.7m Na_2CO_3 + 0.6m NaF	La	12	C_i/na

bdl: solubility below detection limit using the amplitude of the absorption edge from transmission spectra (~ 10 ppm).

na: EXAFS analysis could not be conducted due to low concentration in the high P-T fluids

C_i: The concentration of REE in the high P-T fluids could be estimated (eH values) or calculated (ppm).

Table S2: Comparison of different fitted structures for Yb in alkaline solutions at 200°C. The changing parameters are underlined in bold.

O1-shell			C1-shell			REE2-shell			O2-shell					
N	R	σ^2	N	R	σ^2	N	R	σ	N	R	σ^2	ΔE_0 (eV)	*Rfactor*	χ_{red}
0.7m Na_2CO_3														
8.1(4)	2.30(1)	0.012(1)	2.0(8)	2.75(4)	0.005*	**3.4(2)**	3.87(3)	0.005*	5.6(17)	4.23(4)	0.005*	3.9(5)	0.015	329
8.1(3)	2.31(1)	0.012(1)	2.1(9)	2.75(3)	0.006(5)	**2.4(7)**	3.85(2)	0.001(3)	5.5(23)	4.14(2)	0.003(3)	4.1(3)	0.014	316
8.1(4)	2.30(1)	0.011(1)	2.4(8)	2.72(6)	0.018(1)				**12**	4.10(2)	0.012(5)	3.2 (3)	0.035	576
0.7m Na_2CO_3 - 0.3mNaF														
8.2(3)	2.30(1)	0.010(1)	2.2(6)	2.75(3)	0.005*	**3.8(13)**	3.87(3)	0.005*	6.3(16)	4.13(4)	0.005*	3.6(3)	0.0207	70
8.3(3)	2.30(1)	0.011(1)	2.3(4)	2.75(3)	0.008(5)	**2.4(8)**	3.86(3)	0.0015*	7.6(17)	4.12(3)	0.007(5)	3.4(3)	0.0184	55

Table S3: Coordinates of $[Gd_3(OH)_4(CO_3)^2(H_2O)_{12}]^+$

	x	y	z
Gd	0	0	0
Gd	3.577647	0.439952	-1.221649
Gd	-3.399001	-1.279914	0.200113
O	-1.32536	-2.008609	-0.34156
C	-0.996575	2.626019	0.717728
C	1.017952	-1.897509	1.976354
O	2.13688	1.215915	0.360951
O	0.026023	-1.04202	2.211696
O	-0.221935	2.434263	-0.329635
O	1.485409	-2.676851	2.837363
O	-2.214443	0.242199	-0.991912
O	-1.566584	3.718179	0.980912
O	-1.143211	1.542877	1.502381
O	1.455906	-1.845093	0.717149
H	-0.906757	-2.774485	0.094091
H	1.659255	2.065578	0.207452
H	-2.479651	1.179195	-1.049524
O	1.44424	-0.235895	-1.914153
H	1.301611	-1.006139	-2.496593
O	-3.532825	0.653707	1.733472
H	-2.598012	1.118703	1.707033
H	-4.150435	1.311197	1.355268
O	-5.249011	0.300426	-0.659988
H	-5.16057	0.895737	-1.429118
H	-6.203666	0.289674	-0.455242
O	-3.733857	-3.829164	0.355489
H	-4.327166	-4.222483	1.024949
H	-3.027149	-4.489501	0.219181
O	-2.436497	-1.883298	2.435909
H	-2.447231	-2.842116	2.622457
H	-1.442155	-1.626502	2.446627
O	-4.395002	-2.250349	-1.864151
H	-4.641131	-3.191634	-1.755703
H	-5.177304	-1.8185	-2.260826
O	-5.472392	-1.832631	1.57011
H	-5.622413	-1.324014	2.391695
H	-6.367517	-2.056563	1.249498
O	3.723894	-1.735889	-0.263124
H	2.810855	-1.903082	0.26621
H	4.445542	-2.047641	0.314555

O	6.052993	-0.129242	-1.524665
H	6.671659	0.627682	-1.497343
H	6.519389	-0.846173	-1.052366
O	4.14634	-0.396038	-3.517378
H	3.584717	-0.953343	-4.090626
H	5.03935	-0.794928	-3.555778
O	2.386885	2.03722	-2.812757
H	1.693054	1.317152	-2.831418
H	2.704907	2.115058	-3.734159
O	4.882456	2.553538	-1.366949
H	4.466031	3.340152	-1.772568
H	5.171031	2.844953	-0.476983
O	4.62038	1.111038	1.045902
H	4.971308	0.487398	1.710664
H	3.658275	1.266902	1.278512
